# In vivo glucose metabolism and glutamate levels in mGluR5 knockout mice: a multimodal neuroimaging study using [^18^F]FDG microPET and MRS

**DOI:** 10.1186/s13550-020-00716-z

**Published:** 2020-10-02

**Authors:** Yo-Han Joo, Yun-Kwan Kim, In-Gyu Choi, Hyeon-Jin Kim, Young-Don Son, Hang-Keun Kim, Paul Cumming, Jong-Hoon Kim

**Affiliations:** 1 Neuroscience Research Institute, Gachon University, Incheon, Republic of Korea; 2 Department of Biomedical Sciences, Seoul National University, Seoul, Republic of Korea; 3 Department of Radiology, Seoul National University Hospital, Seoul, Republic of Korea; 4 Department of Biomedical Engineering, College of Health Science, Gachon University, Incheon, Republic of Korea; 5 Gachon Advanced Institute for Health Science and Technology, Graduate School, Gachon University, Incheon, South Korea; 6 Institute of Nuclear Medicine, Inselspital, Bern University, Bern, Switzerland; 7 School of Psychology and Counselling, Queensland University of Technology, Brisbane, Australia; 8 Department of Psychiatry, Research Center for Psychiatry and Behavioral Sciences, Neuroscience Research Institute, Gachon University College of Medicine, Gil Medical Center, Gachon University, 1198 Guwol-dong, Namdong-gu, Incheon, 405-760 South Korea

**Keywords:** mGluR5, Knockout, NMDA, Glutamate, FDG, PET, MRS

## Abstract

**Background:**

Perturbed functional coupling between the metabotropic glutamate receptor-5 (mGluR5) and N-methyl-d-aspartate (NMDA) receptor-mediated excitatory glutamatergic neurotransmission may contribute to the pathophysiology of psychiatric disorders such as schizophrenia. We aimed to establish the functional interaction between mGluR5 and NMDA receptors in brain of mice with genetic ablation of the mGluR5.

**Methods:**

We first measured the brain glutamate levels with magnetic resonance spectroscopy (MRS) in mGluR5 knockout (KO) and wild-type (WT) mice. Then, we assessed brain glucose metabolism with [^18^F]fluorodeoxyglucose ([^18^F]FDG) positron emission tomography before and after the acute administration of an NMDA antagonist, MK-801 (0.5 mg/kg), in the same mGluR5 KO and WT mice.

**Results:**

Between-group comparisons showed no significant differences in [^18^F]FDG standardized uptake values (SUVs) in brain of mGluR5 KO and WT mice at baseline, but widespread reductions in mGluR5 KO mice compared to WT mice after MK-801 administration (*p* < 0.05). The baseline glutamate levels did not differ significantly between the two groups. However, there were significant negative correlations between baseline prefrontal glutamate levels and regional [^18^F]FDG SUVs in mGluR5 KO mice (*p* < 0.05), but no such correlations in WT mice. Fisher’s Z-transformation analysis revealed significant between-group differences in these correlations (*p* < 0.05).

**Conclusions:**

This is the first multimodal neuroimaging study in mGluR5 KO mice and the first report on the association between cerebral glucose metabolism and glutamate levels in living rodents. The results indicate that mGluR5 KO mice respond to NMDA antagonism with reduced cerebral glucose metabolism, suggesting that mGluR5 transmission normally moderates the net effects of NMDA receptor antagonism on neuronal activity. The negative correlation between glutamate levels and glucose metabolism in mGluR5 KO mice at baseline may suggest an unmasking of an inhibitory component of the glutamatergic regulation of neuronal energy metabolism.

## Introduction

Glutamate is the most abundant excitatory neurotransmitter in the brain and is consequently a major driver for neuronal energy expenditure. The energy budget of brain is uniquely dependent on oxidative phosphorylation of pyruvate derived from glycolysis [1, 2]. Nonetheless, neuronal energy metabolism can be assessed in vivo by using positron emission tomography (PET) with the glycolysis tracer [^18^F]fluorodeoxyglucose ([^18^F]FDG), which gives an index of the glucose metabolism that is coupled to glutamatergic neuronal activity [3, 4]. Glutamatergic signaling plays critical roles in synaptic plasticity and the regulation of cognitive, behavioral, and affective processes [5–7]. As such, dysfunction of glutamatergic neurotransmission is implicated in the pathophysiology of major psychiatric disorders such as schizophrenia and depressive disorders [8, 9] and is also involved in excitotoxic and neurodegenerative diseases [10, 11].

The glutamate receptors in brain belong to two major classes, the ionotropic ligand-gated ion channels such as N-methyl-d-aspartate (NMDA) receptors and a diverse family of metabotropic glutamate receptors (mGluR) linked to intracellular second messenger systems. Among the latter group, the type 5 metabotropic receptor (mGluR5) is an important player in glutamatergic signaling [12–14]. Previous studies reported that mGluR5 is functionally linked to NMDA receptors via scaffold proteins of the post-synaptic density (PSD), such as Homer, Shank, and PSD-95 [15, 16]. Increasing evidence indicates that the activation of NMDA receptors is potentiated by concomitant mGluR5-mediated signaling [16, 17] and that stimulation of NMDA receptors reciprocally enhances mGluR5 function [18, 19]. The synergistic action of glutamate signaling at mGluR5/NMDA receptors [20, 21] is further attested by observations that genetic ablation of mGluR5 interfered with NMDA receptor-mediated long-term potentiation in the hippocampus [22, 23]. In addition, while pharmacological antagonism of mGluR5 did not provoke deficits in the prepulse inhibition (PPI) of the acoustic startle response in rodents, mGluR5 antagonism significantly potentiated the PPI disruption induced by the NMDA receptor antagonist, MK-801 [24], suggesting a mGluR5/NMDA synergism in this important behavioral endophenotype of schizophrenia. Furthermore, recent rodent studies associate dysregulation of mGluR5/NMDA signaling with a range of neurocognitive deficits, which are among the core features of schizophrenia [25, 26]. Thus, the further investigation of mGluR5/NMDA synergism may shed new light on the underlying characteristics of glutamatergic dysfunction in psychiatric disorders such as schizophrenia.

The mGluR5 knockout (KO) mouse model enables the investigation of behavioral phenotypes associated with metabotropic glutamatergic signaling abnormalities [20, 27]. However, there has been no direct evaluation of mGluR5/NMDA synergism based on observations of brain energy metabolism and glutamate levels in brain of mice with genetic ablation of the mGluR5. Thus, we undertook this in vivo multimodal imaging study with groups of wild-type (WT) and mGluR5 KO mice to examine the effects of mGluR5 KO on brain glucose metabolism measured with [^18^F]FDG PET and glutamate levels measured with magnetic resonance spectroscopy (MRS). To test the reciprocal interaction between mGluR5 and NMDA receptors, we measured brain glucose metabolism before and after acute administration of the NMDA antagonist MK-801 in WT and KO mice.

## Materials and methods

### Animals

Mice heterozygous for mGluR5 (B6.129-Grm5tm1Rod/J, stock #003558) in C57BL/6J background were purchased from Jackson Laboratory (Bar Harbor, Maine). Three pairs of the first-generation mGluR5 heterozygous mice were bred to obtain 11 mGluR5 KO (Grm5 −/−, six F3 and five F4) and 10 WT (Grm5 +/+, six F3 and four F4) mice. The genotype of each mouse was identified by polymerase chain reaction (PCR) analysis (Macrogen, South Korea). The mice had ad libitum access to food and water and were separately housed in single-sex cages with a 12-h light–dark cycle. All experiments were approved by the Institutional Animal Care and Use Committee (IACUC) of Gachon University (GU-NRI-17-001) and Seoul National University College of Medicine (SNU-16-0189-S1A1) and were carried out in strict accordance with the recommendations in the Guide for the Care and Use of Laboratory Animals 8th edition, National Research Council (2011).

### [^18^F]FDG microPET

The [^18^F]FDG microPET study was performed at Gachon University College of Medicine. Prior to PET scans, each mouse was deprived of food for at least 12 h and then received a bolus tail vein injection of 4.2 ± 0.6 MBq [^18^F]FDG in approximately 200 µL. Baseline PET scans were followed 2 weeks later by second fasting PET scans with acute administration of MK-801 (0.5 mg/kg) at 30 min before [^18^F]FDG injection. Immediately after tracer injection, mice were placed in a holding cage for 30 min of [^18^F]FDG uptake and then rapidly anesthetized with 2.5% isoflurane in 80% oxygen. Within a minute of induction, mice were placed in the aperture of the Focus 120 microPET (Concorde Microsystems, Knoxville, TN, USA) with continued inhalation anesthesia during the recording of a single emission frame lasting 30 min. After attenuation correction, the static PET emission images were reconstructed using a three-dimensional ordered subset expectation maximization (3D-OSEM) algorithm. The voxel size was 0.216 × 0.216 mm and of 0.796 mm thickness. The whole brain PET image of each mouse was segmented from the original PET images by manual editing of the extracerebral voxels using the PMOD software (PMOD version 3.9, PMOD Technologies Ltd., Zurich, Switzerland). The images were then co-registered and spatially normalized to a [^18^F]FDG PET mouse brain template [28] using nine parameters with statistical parametric mapping 12 (SPM12, Wellcome Trust Centre for Neuroimaging, https://www.fil.ion.ucl.ac.uk/spm) as implemented in the PMOD software. For analysis of [^18^F]FDG PET recordings, we calculated standardized uptake values (SUVs) for the baseline scans (SUV_pre_) and MK-801 challenge scans (SUV_post_) using the formula: SUV = tissue activity concentration × body weight/injected radiotracer dose. We calculated mean SUVs in the striatum, cortex, hippocampus, thalamus, cerebellum, and whole brain [29, 30] using regions defined by the mouse region-of-interest (ROI) template [28]. We next calculated the percent change of SUV with the administration of MK-801 in the mGluR5 KO and WT mouse groups as follows: $\Delta {\text{SUV}}\left( \% \right) = \frac{{{\text{SUV}}_{{{\text{post}}}} - {\text{SUV}}_{{{\text{pre}}}} }}{{{\text{SUV}}_{{{\text{pre}}}} }} \times 100$.

### Magnetic resonance spectroscopy

The MRS experiment was performed at Seoul National University College of Medicine approximately two weeks before the PET experiments. For MR data acquisition, mice were anesthetized with isoflurane (1.5% in oxygen) and placed inside the magnet in the prone position. The respiration rate and body temperature of the animals were monitored during the MR scan. All MR data were collected on a 9.4T animal MR scanner with a single-channel surface coil for RF transmission and signal reception (Agilent Technologies, Santa Clara, CA, USA). For voxel localization, scout images were acquired along the axial, coronal, and sagittal directions using a T2-weighted fast spin echo sequence (repetition time (TR)/echo time (TE) = 3000/33 ms, echo train length = 4, field of view (FOV) = 20 × 20 mm^2^, matrix size = 256 × 256, slice thickness = 1 mm, 15 slices for the axial and sagittal directions and 10 slices for the coronal direction (no gap), receiver bandwidth (BW) = 100 kHz, and number of signal averages (NSA) = 2). Based on the scout images, spectroscopy voxels were positioned in the prefrontal cortex (1.5 × 1.5 × 3.0 mm^3^) and hippocampus (1.5 × 2.0 × 2.5 mm^3^). Then, the first- and second-order auto-shimming was performed followed by manual refinement.

^1^H-MRS data were acquired with a spin echo, full-intensity acquired localized (SPECIAL) sequence [31]. To minimize voxel displacement, the carrier frequency was adjusted by − 2.3 ppm from the water resonance. A variable pulse power and optimized relaxation delays (VAPOR) [32] module combined with outer volume suppression (OVS) modules [33] preceded the SPECIAL sequence except for the acquisition of the double inversion recovery (DIR)-based metabolite-nulled spectra. Other sequence parameters were: TR/TE = 4000/2.84 ms, number of data points = 2048, spectral BW = 5 kHz, 32-step phase cycling, and 2 dummy scans. A total of 12 spectra each with NSA = 64 were consecutively acquired for each voxel. A metabolite-nulled spectrum was acquired for each voxel as a surrogate of spectral baseline [34] using a DIR-SPECIAL sequence (TR/TE = 4650/2.84 ms, NSA = 320, and 1st/2nd inversion time (TI) = 2150/686 ms).

The spectral basis set for metabolites was simulated using GAMMA [35] in response to a single spin echo sequence for metabolites, according to the reported chemical shifts and J-coupling constants [36]. Data were zero-filled to 4096 points, apodized, and phase-corrected by using jMRUI [37]. The residual water signal was removed by the HLSVD filter [38]. The individual metabolites were quantified from the spectra using AMARES [39]. The metabolite-nulled spectrum was also incorporated into the spectral basis set [34]. Only the glutamate data with a Cramer–Rao lower bound (CRLB) of ≤ 30% were included in the statistical analysis.

### Statistical analysis

Between-group comparisons of [^18^F]FDG PET SUVs were performed using a linear mixed model. The mouse group and brain ROIs were treated as fixed effects and brain regions were also treated as random effects, while SUV_pre_, SUV_post_ and ΔSUV were treated as dependent variables. We additionally applied the multivariate approach using a repeated measures analysis of variance (ANOVA) of the SUV with time (pre- versus post-MK-801) as a within-subjects factor and group (mGluR5 KO versus WT) as a between-subjects factor, to account for interaction effects of MK-801 treatment and genotype. Given the small sample size, pairwise comparisons between SUV_pre_ and SUV_post_ were also performed using the Wilcoxon rank test. Between-group comparisons of glutamate levels were performed using the Mann–Whitney *U* test. Correlation analyses between baseline regional (prefrontal cortex and hippocampus) glutamate levels and SUV_pre_ were performed in a multimodal PET-MRS approach. To compare between-group differences of the correlation coefficients, Fisher’s Z-transformation analysis was conducted. The significance level was set at *p* < 0.05 for all statistical analyses. All statistical analyses were performed using R software (version 3.5.1, R Foundation for Statistical Computing, Vienna, Austria).

## Results

**Fig. 1:**
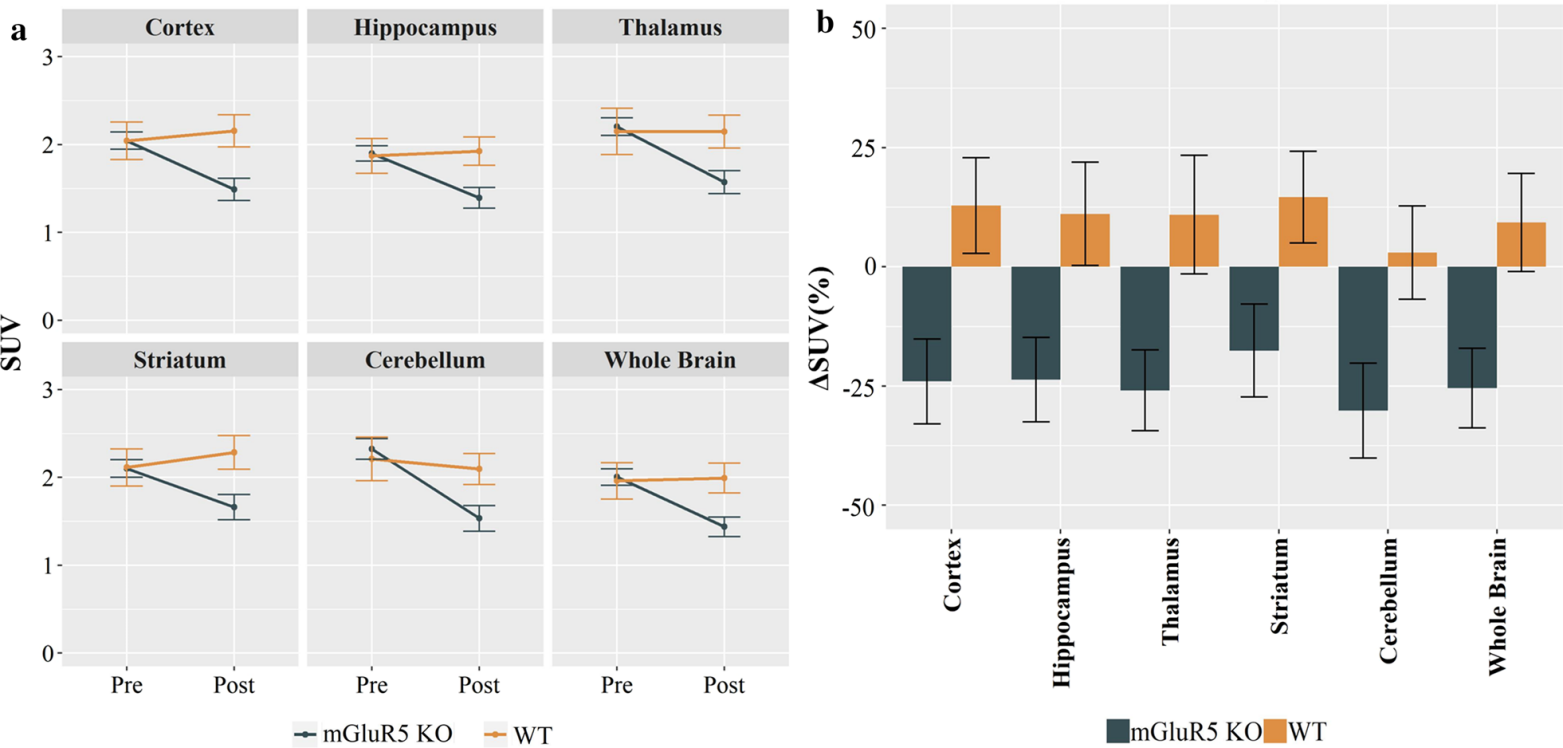
The mean [^18^F]FDG PET SUV and ΔSUV with standard error bars in mGluR5 KO and WT mice. **a** Significantly decreased SUVs were observed in mGluR5 KO mice after the acute administration of MK-801 (*p* < 0.05), whereas no significant alterations were observed in WT mice (*p* > 0.05). **b** The ΔSUV values were significantly different between mGluR5 KO and WT mice in ROIs (*p* < 0.05). SUV, standardized uptake value; ΔSUV, the percent change of SUV; ROI, region of interest

The demographic characteristics of the mGluR5 KO and WT mice are presented in Table 1. Between-group differences of age, sex, weight, and injected [^18^F]FDG dose were not significant (Table 1). The SUV_pre_ and SUV_post_ are shown in Fig. 1a, and the ΔSUV is shown in Fig. 1b for the six ROIs (Fig. 1).

**Table 1:** Demographic characteristics and scan parameters

Variables	Mouse type		*t-*value	*p*-value
	mGluR5 KO (*n* = 11) Mean ± SD	WT (*n* = 10) Mean ± SD		
Age (day)	252.6 ± 68.5	222.4 ± 45.9	1.66	0.10
Male/female	8/3	5/5	0.39^†^	0.39
Weight (g)	22.9 ± 4.2	24.0 ± 3.5	− 0.93	0.36
[^18^F] FDG injected dose (MBq)	4.1 ± 0.6	4.3 ± 0.6	− 1.02	0.32
[^18^F] FDG injected dose/weight (MBq/g)	0.18 ± 0.37	0.18 ± 0.32	0.20	0.84

*KO* knockout, *WT* wild type, *SD* standard deviation

^†^Odds ratio

### Between-group comparisons

The linear mixed model analysis of ΔSUV as the dependent variable showed a significant main effect of group [F(1,19) = 6.62, *p* = 0.019], and the linear mixed model analysis of SUV_post_ as the dependent variable also showed a significant main effect of group [F(1,19) = 7.08, *p* = 0.015]. However, the analysis of SUV_pre_ as the dependent variable showed no significant main effect of group [F(1,19) = 0.05, *p* = 0.827]. The Bonferroni post hoc analysis revealed significantly lower SUV_post_ and ΔSUV in all ROIs in the mGluR5 KO mice compared to WT mice (*p* < 0.05), whereas there were no significant between-group differences in SUV_pre_ in any ROI (*p* > 0.1) (Fig. 1, Table 2). The repeated measures ANOVA also showed a significant MK-801 treatment x genotype interaction effect for all ROIs [cortex: F(1,19) = 5.91, *p* = 0.039; hippocampus: F(1, 19) = 4.79, *p* = 0.041; thalamus: F(1, 19) = 4.68, *p* = 0.043; striatum: F(1, 19) = 4.45, *p* = 0.048, cerebellum: F(1, 19) = 4.42, *p* = 0.049; whole brain: F(1, 19) = 4.90, *p* = 0.039]. Additional pairwise comparisons using the Wilcoxon rank test showed significantly decreased SUVs after administration of MK-801 in all ROIs in the mGluR5 KO mice (*p* < 0.05), except for the striatum (*p* = 0.054), but no significant changes of SUVs in WT mice (*p* > 0.1) (Table 3). Among the 11 mGluR5 KO mice, the glutamate levels were available in the prefrontal cortex of nine mice and the hippocampus of ten mice, while MRS for glutamate was successful in all WT mice in both regions. Between-group comparisons using the Mann–Whitney *U* test showed no significant differences in glutamate levels (*p* > 0.1) (Table 4).

**Table 2:** Between-group comparisons of [^18^F]FDG PET SUV_pre_, SUV_post_ and ΔSUV with acute MK-801 challenge

Region	Group	SUV_pre_ Mean ± SD	*t*	*p*-value	SUV_post_ Mean ± SD	*t*	*p*-value	ΔSUV (%) Mean ± SD	*t*	*p*-value
CTX	mGluR5 KO	2.04 ± 0.33	0.00	0.998	1.49 ± 0.42	− 3.00	0.007**	− 24.0 ± 29.5	− 2.64	0.016*
	WT	2.04 ± 0.68			2.16 ± 0.58			12.8 ± 31.8		
HIP	mGluR5 KO	1.90 ± 0.29	0.11	0.912	1.39 ± 0.40	− 2.40	0.027*	− 23.6 ± 29.4	− 2.49	0.022*
	WT	1.87 ± 0.63			1.93 ± 0.51			11.1 ± 34.2		
THA	mGluR5 KO	2.20 ± 0.34	0.22	0.825	1.57 ± 0.43	− 2.60	0.018*	− 25.9 ± 28.1	− 2.64	0.016*
	WT	2.15 ± 0.83			2.15 ± 0.60			11.0 ± 39.4		
STR	mGluR5 KO	2.10 ± 0.33	− 0.05	0.961	1.66 ± 0.47	− 2.81	0.011*	− 17.5 ± 32.4	− 2.30	0.033*
	WT	2.11 ± 0.67			2.29 ± 0.61			14.7 ± 30.4		
CB	mGluR5 KO	2.32 ± 0.39	0.47	0.643	1.53 ± 0.49	− 2.54	0.020*	− 30.1 ± 33.0	− 2.37	0.028*
	WT	2.21 ± 0.79			2.10 ± 0.56			3.0 ± 30.9		
WB	mGluR5 KO	2.00 ± 0.31	0.20	0.845	1.44 ± 0.37	− 2.79	0.011*	− 25.4 ± 27.6	− 2.65	0.016*
	WT	1.96 ± 0.66			1.99 ± 0.53			9.3 ± 32.4		

*SUV* standardized brain uptake value for [^18^F]FDG, *ΔSUV* the percent change of SUV, *SD* standard deviation, *KO* knockout, *WT* wild type, *CTX* cortex, *HIP* hippocampus, *THA* thalamus, *STR* striatum, *CB* cerebellum, *WB* whole brain

^*^p < 0.05, **p < 0.01

**Table 3:** Wilcoxon rank test results for pairwise comparisons in mGluR5 KO and WT mice

Group	Region	SUV_pre_ Mean ± SD	SUV_post_ Mean ± SD	*W*	*p* value
mGluR5 KO	CTX	2.04 ± 0.33	1.49 ± 0.42	8	0.024*
	HIP	1.90 ± 0.29	1.39 ± 0.40	8	0.024*
	THA	2.20 ± 0.34	1.57 ± 0.43	6	0.014*
	STR	2.10 ± 0.33	1.66 ± 0.47	11	0.054
	CB	2.32 ± 0.39	1.53 ± 0.49	5	0.010**
	WB	2.00 ± 0.31	1.44 ± 0.37	6	0.014*
WT	CTX	2.04 ± 0.68	2.16 ± 0.58	38	0.322
	HIP	1.87 ± 0.63	1.93 ± 0.51	37	0.375
	THA	2.15 ± 0.83	2.15 ± 0.60	30	0.846
	STR	2.11 ± 0.67	2.29 ± 0.61	41	0.193
	CB	2.21 ± 0.79	2.10 ± 0.56	29	0.922
	WB	1.96 ± 0.66	1.99 ± 0.53	34	0.557

*KO* knockout, *WT* wild type, *SUV* standardized uptake value, *SD* standard deviation, *CTX* cortex, *HIP* hippocampus, *THA* thalamus, *STR* striatum, *CB* cerebellum, *WB* whole brain

^*^p < 0.05, **p < 0.01

**Table 4:** Between-group comparisons of glutamate levels measured using magnetic resonance spectroscopy

	Region	Group	Mean ± SD	*W*	*p* value	Effect size
Glutamate	HIP	mGluR5 KO	0.70 ± 0.11	58	0.579	0.135
		WT	0.69 ± 0.13			
	PFC	mGluR5 KO	1.02 ± 0.17	37	0.549	0.150
		WT	1.14 ± 0.42			

*KO* knockout, *WT* wild type, *PFC* prefrontal cortex, *HIP* hippocampus

### Correlation analyses

**Fig. 2:**
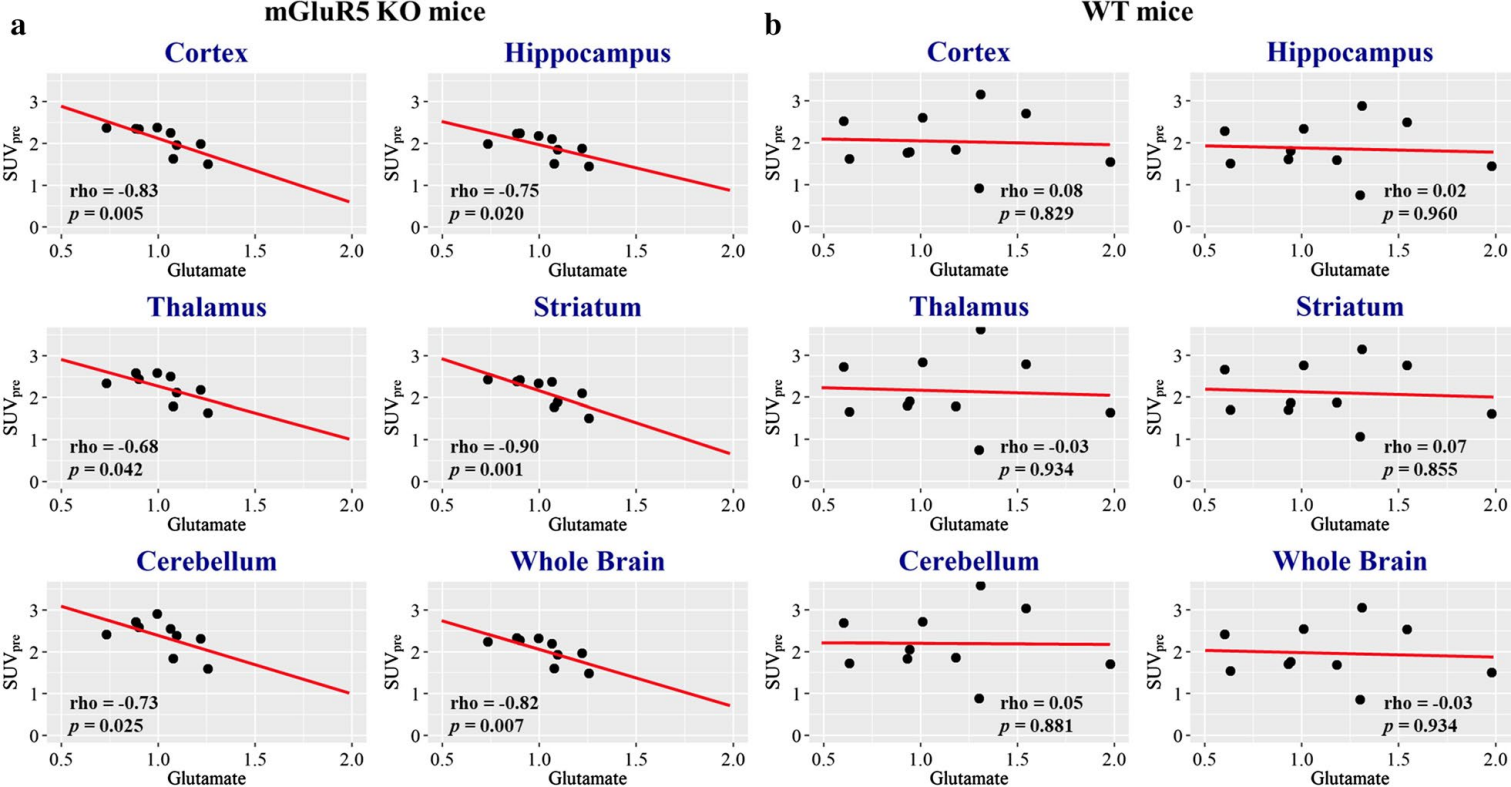
Correlation analysis results between prefrontal glutamate levels to MRS and the baseline [^18^F]FDG PET results (SUV_pre_) in ROIs. There were significant negative correlations in mGluR5 KO mice (*p* < 0.05) (**a**), while no significant correlations were observed in WT mice (**b**). Fisher’s Z-transformation showed significant between-group differences of correlation coefficients in the cortex (*z* = − 2.30 *p* = 0.022), striatum (*z* = − 2.77 *p* = 0.006), and whole brain (*z* = − 2.01 *p* = 0.045). SUV, standardized uptake value; ROI, region of interest

The correlation analysis showed significant negative correlations between baseline glutamate levels in the prefrontal cortex and SUV_pre_ in the six ROIs in mGluR5 KO mice: cortex (rho = − 0.83, *p* = 0.005), striatum (rho = − 0.90, *p* = 0.001), hippocampus (rho = − 0.75, *p* = 0.020), thalamus (rho = − 0.68, *p* = 0.042), cerebellum (rho = − 0.73, *p* = 0.025), and whole brain (rho = − 0.82, *p* = 0.007) (Table 5, Fig. 2). However, there were no significant correlations between prefrontal glutamate levels and SUV_pre_ in WT mice (*p* > 0.1) (Table 5, Fig. 2). The Fisher’s Z-transformation analysis revealed significant between-group differences of correlation coefficients between glutamate levels in the prefrontal cortex and SUV_pre_ in the three ROIs as follows: cortex (*z* = − 2.30, *p* = 0.022), striatum (*z* = − 2.77, *p* = 0.006), and whole brain (*z* = − 2.01, *p* = 0.045) (Table 5, Fig. 2). There were no significant correlations between baseline hippocampal glutamate levels and SUV_pre_ in either mGluR5 KO or WT mice (*p* > 0.1) (Table 5).

**Table 5:** Correlation coefficients between cortical glutamate levels and baseline [^18^F]FDG PET results (SUV_pre_) with Fisher’s Z-transformation

MRS region	PET region	mGluR5 KO			WT			Fisher’s *Z*	*p *value
		*n*	Rho	*p* value	*n*	Rho	*p* value		
PFC	CTX	9	− 0.83	0.005**	10	0.08	0.829	− 2.30	0.022*
	HIP	9	− 0.75	0.020*	10	0.02	0.960	− 1.78	0.075
	THA	9	− 0.68	0.042*	10	− 0.03	0.934	− 1.45	0.148
	STR	9	− 0.90	0.001***	10	0.07	0.855	− 2.77	0.006**
	CB	9	− 0.73	0.025*	10	0.05	0.881	− 1.78	0.075
	WB	9	− 0.82	0.007**	10	− 0.03	0.934	− 2.01	0.045*
HIP	CTX	10	0.09	0.803	10	0.09	0.803	0.00	1.000
	HIP	10	0.19	0.603	10	0.18	0.627	0.02	0.982
	THA	10	0.05	0.881	10	0.24	0.511	− 0.35	0.729
	STR	10	0.27	0.446	10	0.04	0.907	0.45	0.656
	CB	10	0.13	0.726	10	0.15	0.676	− 0.05	0.962
	WB	10	0.03	0.934	10	0.24	0.511	− 0.51	0.613

*SUV* standardized uptake value, *KO* knockout, *WT* wild type, *PFC* prefrontal cortex, *CTX* cortex, *HIP* hippocampus, *THA* thalamus, *STR* striatum, *CB* cerebellum, *WB* whole brain

^*^p < 0.05, **p < 0.01, ***p < 0.001

## Discussion

We examined the effects of mGluR5 genetic deletion on cerebral glucose metabolism measured with [^18^F]FDG PET and glutamate levels measured using MRS in living mice. While glutamate levels were measured only at baseline, we measured [^18^F]FDG PET uptake before and after acute administration of the potent NMDA antagonist MK-801, aiming to probe the interaction between mGluR5 and NMDA receptors by regarding cerebral glucose metabolism as a proxy for net neuronal activity. At baseline, there were no significant group differences in brain glucose metabolism between mGluR5 KO and WT mice. However, we observed significantly lower glucose metabolism in the MK-801 challenge condition relative to baseline in the mGluR5 KO mice. There were significant negative correlations between prefrontal glutamate levels and regional glucose metabolism in mGluR5 KO mice, but no such correlations in WT mice, nor did glutamate levels differ between the mGluR5 KO and WT mice at baseline. Thus, treatment with the NMDA antagonist perturbed cerebral [^18^F]FDG uptake only in the mGluR5 KO mice. This observation of an interaction between mGluR5 genetic deletion and acute MK-801 administration resulting in net global hypometabolism seems to be in line with the reported reciprocal potentiation between glutamate signaling at mGluR5 and NMDA receptors [20, 21].

In our study, there was no statistically significant effect of acute MK-801 treatment on cerebral [^18^F]FDG uptake in WT mice. A subanesthetic dose of the NMDA antagonist ketamine increased [^18^F]FDG uptake in some rat brain regions [40]. Similarly, previous rodent studies reported focal increases in [^18^F]FDG trapping in retrosplenial and posterior cingulate cortices after a single dose of MK-801 (0.4 mk/kg) [41] and decreased [^18^F]FDG uptake after one week of daily MK-801 administration (0.3 mg/kg) [42]. Our finding of a trend toward a global 10% increase in [^18^F]FDG uptake (Fig. 1) in the WT group was obscured by the high relative standard deviation in the baseline evaluation of SUV. However, sample size calculation for our finding of a 10% increase in SUV with a 15% relative standard deviation predicts that we would have needed much larger populations of mice to avoid a type II statistical error.

Previous microdialysis studies showed that MK-801 treatment acutely increases extraneuronal glutamate levels in rodent brain [43–45], which has been attributed to a net disinhibition of glutamate release via blockade of excitatory inputs to gamma-aminobutyric acid (GABA)-ergic interneurons in the cerebral cortex [46, 47]. The present lack of significant effect of MK-801 on cerebral [^18^F]FDG uptake in WT mice suggests that any stimulation of glucose consumption via increased activation by endogenous glutamate at mGluR5 and other non-NDMA sites may normally be roughly balanced by a reduced drive of neuronal activity arising from acute blockade of the NMDA sites. However, this putative balance was absent in the mGluR5 KO mice after MK-801 challenge, where we observed a global and highly significant 25% reduction in [^18^F]FDG uptake relative to baseline. Assuming the MK-801 treatment indeed increased glutamate release, we suppose that absent mGluR5 signaling resulted in failure to accommodate for the reduced metabolic drive from the blocked NMDA receptors. We suppose that these results have some relevance for excitotoxicity of glutamate in the context of “Olney’s lesion,” which refers to neurovacuolation and mitochondrial damage confined to the posterior cingulate and retrosplenial cortices of rats treated with MK-801 and other NMDA antagonists [48]. Interestingly, these are the same regions showing hypermetabolism on [^18^F]FDG PET in the acute MK-801 rat study by Shirakawa et al. [41] cited above. The present findings thus suggest a testable hypothesis that mGluR5 KO mice should be protected from Olney’s lesion, by virtue of the attenuation of global neuronal activity seen with [^18^F]FDG PET.

In the MRS arm of the study, we observed no significant differences in baseline prefrontal and hippocampal glutamate levels between the WT and mGluR5 KO mice, nor did we see any relationship between individual glutamate levels and regional [^18^F]FDG SUVs in the WT animals. However, there were significant inverse correlations between glutamate levels in the prefrontal cortex and regional [^18^F]FDG uptake in the mGluR5 KO mice. This might imply that mGluR5 KO unmasks a coupling between brain glutamate levels in the prefrontal cortex and global neuronal activity, which is normally obscured in WT mice. We can only speculate how this might arise, but we note that AMP-activated protein kinase (AMPK) phosphorylation is suppressed in mice with genetic ablation of mGluR5 [49]. Given that AMPK is critically involved in the regulation of glycolysis and mitochondrial respiration in response to synaptic activity [50], its homeostatic regulation may be perturbed in mGluR5 KO mice, thus contributing to the emergence of the negative correlation between prefrontal glutamate and regional [^18^F]FDG uptake. There is little precedent for this finding, as there seems to be no previous studies linking glutamate levels with [^18^F]FDG uptake in rodent brain. One report in Alzheimer’s disease patients reported no correlation between the amplitude of the glutamate/glutamine peak and the globally normalized [^18^F]FDG SUV [51]. Another such multimodal study in epilepsy patients showed a significant correlation between the glutamate signal and [^18^F]FDG uptake in the cortex contralateral to the epileptic focus [52]. Thus, there is a clear need for further investigation of these relationships in healthy and diseased brain.

Accumulating evidence suggests that glutamatergic signaling, particularly via NMDA receptors, is altered in schizophrenia [53]. A recent *postmortem* brain study demonstrated a significant attenuation of mGluR5 signaling in the prefrontal cortex from patients with schizophrenia, which was associated with reduced NMDA receptor phosphorylation, suggesting that disruption of reciprocal interaction between mGluR5 and NMDA signaling is integral to glutamatergic dysfunction in schizophrenia [54]. Our present results in mGluR5 KO mice revealed significantly perturbed brain glucose metabolism that emerged only upon administration of the NMDA antagonist, thus implying an interaction of the two types of glutamate receptor in regulating brain glucose metabolism. Along with previous reports that mGluR5 antagonists significantly potentiate MK-801-induced neurocognitive deficits in rats that resemble the cognitive symptoms of schizophrenia [24, 55], our results suggest that the mGluR5s may have little independent effects on brain glucose metabolism other than those mediated by interaction with NMDA receptors. A very recent PET study reported that lower mGluR5 availability in the left temporal cortex and caudate was associated with more severe negative symptoms and worse cognitive performance in male patients with chronic schizophrenia [56]. Given these clinical and preclinical results, it should prove important to assess the relationships between mGluR5 availability, glutamate levels, and [^18^F]FDG uptake in patients with schizophrenia.

We acknowledge some limitations of the present study. The relatively small sample size may have limited the statistical power to detect group differences in the baseline PET and MRS measurements. In WT mice, the relative standard deviation of the means of baseline SUVs and prefrontal glutamate levels exceeded those in mGluR5 KO mice. However, the variances are in the range of those in previous studies [57, 58]. In the MRS arm of the study, we measured only baseline glutamate levels. An additional study arm to measure glutamate levels after acute administration of MK-801 in mGluR5 KO and WT mice might have confirmed our interpretation that absent mGluR5 signaling results in failure to accommodate the reduced metabolic drive from blocked NMDA receptors, in spite of MK-801-induced glutamate release. This additional study would also have clarified the possible coupling/decoupling of cortical glutamate levels and global neuronal activity in mGluR5 KO and WT mice. Given the reported association between dysregulation of the mGluR5/NMDA system with the pathophysiology of schizophrenia, we measured glutamate levels only in the prefrontal cortex and hippocampus, the two cortical regions most critically implicated in the schizophrenia pathophysiology [59]. Future studies may be needed to investigate whether glutamate levels are altered in other brain areas in mGluR5 KO mice. In addition, given the global nature of glutamatergic signaling, investigations of other regions including the striatum and thalamus would be necessary to assess the broader regional correlations between glutamate levels and [^18^F]FDG uptake in mGluR5 KO and WT mice. Since we had no vehicle or placebo group to control for comparisons in our MK-801 challenge experiments, we cannot exclude the possibility of confounds such as differential responses to stress in mGluR5 KO and WT mice. Since we did not measure blood glucose levels, we cannot exclude the possibility that treatment and genotype influenced plasma glucose levels, and thus [^18^F]FDG uptake. However, the previous investigation of a tracer for detecting pancreatic β-cell mass did not show any effects of MK-801 treatment on glucose-stimulated insulin secretion, from which we infer that present results were not due to global effects of plasma glucose in mGluR5 KO [60, 61]. Moreover, the largest MK-801-induced SUV decrease in the KO mice was in the cerebellum (ΔSUV: − 30.1%), whereas the smallest MK-801-induced SUV increase in the WT mice occurred in the cerebellum (ΔSUV: 3.0%). This finding of regional differences in MK-801-induced changes in [^18^F]FDG uptake also argues against peripheral effects.

In conclusion, this is the first report on cerebral glucose metabolism and glutamate levels in living mGluR5 KO mice and the first report on the association between glucose metabolism and glutamate levels in living rodents. We observed no significant group differences in baseline [^18^F]FDG PET measurements or MRS glutamate levels between WT and mGluR5 KO mice. However, there was a substantial decline in [^18^F]FDG SUVs specifically in the mGluR5 KO mice after challenge with an NMDA antagonist, suggesting that mGluR5 transmission normally moderates the net effects of NMDA receptor antagonism on glucose metabolism, i.e., neuronal activity. We also found significant inverse correlations between prefrontal glutamate levels and [^18^F]FDG uptake in widespread brain areas at baseline in the mGluR5 KO mice, suggesting the unmasking of an inhibitory component of the glutamatergic regulation of neuronal energy metabolism. Our study also suggests that multimodal neuroimaging in combination with pharmacological challenge using the mGluR5 KO mouse model may help to elucidate the nature of mGluR5/NMDA receptor interactions in the control of cerebral metabolism, with implications for disorders such as schizophrenia.

## Data Availability

The datasets used and/or analyzed during the current study are available from the corresponding author on request.

## Abbreviations

AMPK: AMP-activated protein kinase
BW: Bandwidth
3D-OSEM: Three-dimensional ordered subset expectation maximization
[^18^F]FDG: [^18^F]fluorodeoxyglucose
FOV: Field of view
GABA: Gamma-aminobutyric acid
IACUC: Institutional Animal Care and Use Committee
KO: Knockout
mGluR5: Metabotropic glutamate receptor-5
MRS: Magnetic resonance spectroscopy
NMDA: N-methyl-d-aspartate
NSA: Number of signal averages
OVS: Outer volume suppression
PCR: Polymerase chain reaction
PET: Positron emission tomography
PPI: Prepulse inhibition
SPECIAL: Spin echo, full-intensity acquired localized
SPM12: Statistical parametric mapping 12
SUV: Standardized uptake value
ΔSUV: The percent change of SUV
TE: Echo time
TR: Repetition time
VAPOR: Variable pulse power and optimized relaxation delays
WT: Wild type
